# A randomised controlled trial of an implementation strategy delivered at scale to increase outdoor free play opportunities in early childhood education and care (ECEC) services: a study protocol for the get outside get active (GOGA) trial

**DOI:** 10.1186/s12889-022-12883-w

**Published:** 2022-03-29

**Authors:** Sze Lin Yoong, Nicole Pearson, Kathryn Reilly, Luke Wolfenden, Jannah Jones, Nicole Nathan, Anthony Okely, Patti-Jean Naylor, Jacklyn Jackson, Luke Giles, Noor Imad, Karen Gillham, John Wiggers, Penny Reeves, Kate Highfield, Melanie Lum, Alice Grady

**Affiliations:** 1 School of Health Sciences, Department of Nursing and Allied Health, Swinburne University of Technology, Hawthorn, VIC 3122 Australia; 2 School of Medicine and Public Health, College of Health, Medicine and Wellbeing, University of Newcastle, University Drive, Callaghan, NSW 2308 Australia; 3 Hunter New England Population Health, Wallsend, NSW 2287 Australia; 4 Hunter Medical Research Institute, New Lambton Heights, NSW 2305 Australia; 5 Priority Research Centre for Health Behaviour, University of Newcastle, University Drive, Callaghan, NSW 2308 Australia; 6 Early Start, Faculty of the Arts, Social Sciences and Humanities, University of Wollongong, Wollongong, NSW 2522 Australia; 7 School of Health and Society, Faculty of the Arts, Social Sciences and Humanities, University of Wollongong, Wollongong, NSW 2522 Australia; 8 Illawarra Health and Medicine Research Institute, Wollongong, NSW 2522 Australia; 9 School of Exercise Science, Physical and Health Education, University of Victoria, Mackinnon 120, PO Box 1700, STN CSC, Victoria, BC V8W 2Y2 Canada; 10 Early Childhood Australia, Canberra, ACT Australia

**Keywords:** Outdoor play, Free play, Physical activity, Indoor-outdoor, Early childhood education and care, Implementation trial, Randomised controlled trial

## Abstract

**Background:**

Increased outdoor play time in young children is associated with many health and developmental benefits. This study aims to evaluate the impact of a multi-strategy implementation strategy delivered at scale, to increase opportunities for outdoor free play in Early Childhood Education and Care (ECEC) services.

**Methods:**

The study will employ a parallel-group randomised controlled trial design. One hundred ECEC services in the Hunter New England region of New South Wales, Australia, will be recruited and randomised to receive either a 6-month implementation strategy or usual care. The trial will seek to increase the implementation of an indoor-outdoor routine (whereby children are allowed to move freely between indoor and outdoor spaces during periods of free play), to increase their opportunity to engage in outdoor free play. Development of the strategy was informed by the Behaviour Change Wheel to address determinants identified in the Theoretical Domains Framework. ECEC services allocated to the control group will receive ‘usual’ implementation support delivered as part of state-wide obesity prevention programs. The primary trial outcome is the mean minutes/day (calculated across 5 consecutive days) of outdoor free play opportunities provided in ECEC services measured at baseline, 6-months (primary end point) and 18-months post baseline. Analyses will be performed using an intention-to-treat approach with ECEC services as the unit of analysis, using a linear mixed effects regression model to assess between-group differences. A sensitivity analysis will be undertaken, adjusting for service characteristics that appear imbalanced between groups at baseline, and a subgroup analysis examining potential intervention effect among services with the lowest baseline outdoor free play opportunities.

**Discussion:**

Identifying effective strategies to support the implementation of indoor-outdoor routines in the ECEC setting at scale is essential to improve child population health.

**Trial registration:**

Australian New Zealand Clinical Trials Registry (ACTRN12621000987864). Prospectively registered 27th July 2021, ANZCTR - Registration.

## Background

Outdoor play is recognised as essential for healthy child growth and development [1]. Systematic review evidence suggests that increased outdoor play time in children aged 3–12 years is associated with increased moderate-to-vigorous intensity physical activity (MVPA) and cardiorespiratory fitness, as well as accruing other social, emotional, and cognitive benefits [2, 3]. Building on the time-use epidemiology pertaining to 24-h movement behaviours recommended by international guidelines [4, 5], transitioning some indoor only play time to outdoor play time can provide children with additional opportunity to accumulate the benefits of outdoor play. Despite this, national surveys conducted in the United States (US) [6], Canada and Australia suggest that the amount of outdoor play that young children participate in has declined over the last 20 years [7]. As such, leading international organisations including the US Centres for Disease Control Obesity Centre [8], and the United Kingdom’s (UK) Play Safe Forum [9] have recommended increasing opportunities for child self-directed (‘free’) play outdoors in all settings including home, schools, early childhood education and care (ECEC) services and the community [1].

In many countries, ECEC services provide access to the majority of young children aged 0–5 years for a significant proportion of their waking hours [10]. ECEC services therefore have a crucial role in ensuring sufficient opportunities for outdoor play for young children. Almost all ECEC services have the infrastructure and spaces to create environments to support physically active outdoor play [11]. Furthermore, accreditation processes and best-practice guidelines for the ECEC sector recommend that services implement several evidence-based practices supportive of outdoor free play. This includes creating conducive outdoor environments and allowing children to move freely between indoor and outdoor spaces (also known as indoor-outdoor play) [12]. A systematic review of observational ECEC-based studies reported that young children have higher total physical activity outdoors compared to indoors while in care [13]. Increasing outdoor free play opportunities by one hour increased child total physical activity by 25 min, of which 10.7 min was MVPA [13]. Such findings are supported by a recent randomised controlled trial (RCT) conducted in 10 ECEC services, which found that increasing frequency and/or duration of outdoor free play in ECEC services improved child MVPA [14].

Despite best practice guidelines and evidence supporting the need to increase outdoor free play opportunities in ECEC services [1, 15, 16], the amount of time for child-directed outdoor free play remains low [17]. Recommendations for the ECEC sector specify that children should spend between 12 and 20% of the ECEC day engaged in outdoor free play [18, 19]. Observational studies in the US, however, have found that children in care spend an average of 33 min in outdoor free play, representing just 8% of the ECEC day [15]. A national survey of 203 Australian ECEC services indicated that approximately 60% were currently implementing routines including indoor-outdoor play to increase time spent outdoors [20]. Although the majority of ECEC managers indicate being supportive of outdoor free play and implementing indoor-outdoor routines, they also report the need for assistance with planning appropriate staffing and practical strategies to orientate the layout of their service to facilitate transitions outside [20].

Maximising the potential benefits of outdoor free play on child health requires strategies to support the implementation of routines that are conducive to outdoor play at scale. Scaling up has been defined as “a deliberate process of taking health interventions that have been proven effective on a small scale and expanding their reach to larger number of users in real world settings while maintaining efficacy” [21, 22]. The World Health Organization has identified scaling up of programs as crucial to ensuring benefits at a population level [23]. Despite this, less than 3% of physical activity interventions are successfully implemented at scale and very little is known about how to scale-up physical activity programs [24, 25]. In ECEC services, a recent Cochrane review [26], found only two controlled trials that assessed the impact of strategies to increase the implementation of physical activity (PA) programs at scale (defined as > 50 ECEC services in the intervention arm) [27, 28] providing little information about how to support implementation of evidence-based physical activity programs in ECEC services at scale.

This protocol (version 1) describes the methods for an RCT that aims to assess the impact of a multi-strategy implementation strategy, delivered at scale, to increase opportunities for outdoor free play in ECEC services.

## Methods

The study methods will be reported in accordance with the CONSORT reporting standards for RCTs, [29] the Standards for Reporting Implementation Studies (StaRI) statement [30], and is consistent with best practice guidance for undertaking implementation trials [31]. The trial was prospectively registered with the Australian New Zealand Clinical Trials Registry (ACTRN12621000987864). Ethics approval has been provided by Hunter New England (HNE) Human Research Ethics Committee (HREC) (reference no 2019/ETH12353), Swinburne University of Technology HREC (reference no 20215523–5944) and University of Newcastle HREC (reference no H-2008-0343). This protocol has also been reported according to the Standard Protocol Items: Recommendations for Interventional Trials (SPIRT) [32].

### Study design and setting

**Fig. 1:**
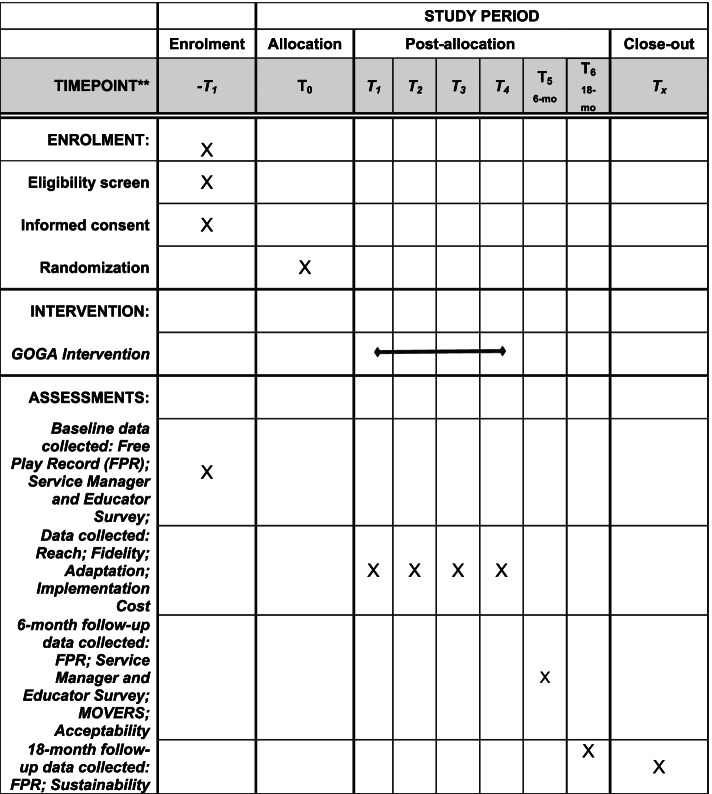
SPIRT Figure

The study will employ a two-arm parallel-group RCT design. One hundred ECEC services in the HNE region of New South Wales (NSW) Australia will be recruited and randomised to receive either a 6-month implementation strategy or usual care. The trial will assess between-group differences in the mean minutes children are provided with the opportunity for outdoor free play per week, with data collected at baseline (approximately November 2021–February 2022), and immediately following the delivery of the 6 month implementation strategy (primary time point, T_5_). Data will also be collected approximately 12-months following completion of the implementation strategy to determine the longer-term impact of the intervention (18-months follow up, T_6_) (Fig. 1).

### Participants and recruitment

#### Sampling frame

A list of all centre-based ECEC services (i.e. long day care and preschool services) located in the HNE Local Health District (LHD) of NSW, Australia (*n* = 440) will be accessed via NSW Centre for Population Health (CPH) records [33] and will serve as the study sampling frame. Within NSW, long day care services provide centre-based care for children from 6 weeks to under 6 years of age for 8 or more hours per day. Preschools typically enrol children between 3 and 6 years of age and provide care for 6 to 8 h per day. The HNELHD area encompasses major metropolitan centres and inner regional communities, with 14% of the population located in remote communities [34].

#### Eligibility

To be eligible to participate, ECEC services must report having at least one session of indoor only free play across five consecutive days, where the opportunity for outdoor free play is not made available to children. Services will be excluded if they are participating in any other trial related to improving physical activity practices, cater exclusively for children with special needs, are an occasional care or family day care service, or are a Department of Education community run service (as such services are not covered within the existing ethics arrangement and consists of less than 10% of services in the HNELHD region).

#### Recruitment procedures

A pre-recruitment telephone call will be undertaken by the research team with all services in the study region to determine eligibility for the study. Following this, all eligible services will be emailed information statements and consent forms outlining study requirements and inviting participation. Recruitment of services will occur in random order and be overseen by an experienced trial coordinator. To maximise study participation, research team members will employ an evidence-based recruitment strategy which includes multiple attempts to contact services with follow up by the same team member, monitoring of consent rates and leveraging of the existing relationships of health service partners [27]. An electronic consent form will be completed by the ECEC Service Manager on behalf of the service to participate in the trial (−T_1_).

### Randomisation and blinding

An independent statistician will set-up block randomisation using a computerised random number function to randomise services in a 1:1 ratio to either the intervention or usual-care control group. Block randomisation (2, 4 or 6) will be used to ensure group allocation is approximately equal. Allocation will be stratified by service type (long day care service or preschool) and service size (small < 80 child enrolments or large > = 80 or more child enrolments) given an association exists between these factors and the implementation of indoor-outdoor routines [20]. Randomisation will be undertaken following baseline data collection (T_0_), and Service Managers will be notified of their group allocation following baseline data collection.

The trial will be conducted as an open trial as it is not possible to deliver the intervention without revealing allocation to ECEC services and those delivering the intervention. Outcome assessors and those undertaking data analysis will remain blinded to service allocation. Outcome assessors will not have access to service allocation data and data analysis will be performed by a statistician not involved in the trial.

### Evidence-based practice (EBP)

The implementation strategy described below seeks to increase the amount of time ECEC services provide children with opportunities for outdoor free play. Increased outdoor free play in childcare is associated with increased child physical activity, supports the development of gross motor skills and has been reported to enhance the quality of educator-child interaction [35]. This practice is aligned with national accreditation standards for the sector as well as state health promotion programs. Currently there are no specific recommendations for amount of time, however best practice guidance specifies that ‘more is better’.

### Implementation strategy

#### Theoretical frameworks

The Get Outside Get Active (GOGA) program is an implementation strategy designed to increase the delivery of the evidence-based practice described above. Primarily, services are supported to modify their play routines to convert any indoor only free play routine to also incorporate outdoor play.

The development of the multi-strategy implementation strategy was informed by the Behaviour Change Wheel (BCW) [36] and Theoretical Domains Framework (TDF) [37]. The TDF summarises 33 theories and 128 constructs which may explain implementation behaviour, into 14 domains underpinned by psychological theory. This framework was used to enable comprehensive barriers assessment to inform intervention development [38]. Barriers to service provision of outdoor free play opportunities were first identified via: i) a systematic review of barriers and facilitators to the implementation of physical activity policies in ECEC [39]; b) quantitative survey with ECEC services to assess key barriers and enablers to providing more outdoor free play opportunities [20]; and c) consultation with Service Managers and Educators from five ECEC services about the key barriers to implementation. The barriers identified from this process are described in Table 1. The capability, opportunity and motivation model of behaviour (COM-B) of the BCW was then used to distil the TDF into these three key domains to enable selection of intervention functions [38].

**Table 1:** Logic model outlining GOGA development

**Proposed mechanisms of action**			**Content**	
Target behaviour: Amending all indoor only free play time to allow children the opportunity to access outdoor areas				
**Barriers reported by ECEC services**	COM-B and (TDF)	Intervention function	Behaviour Change Techniques (BCTs) applied	Implementation strategy (modality)
Staff lack of awareness of the benefits of outdoor play and implementing indoor-outdoor routines.	Capability – psychological (Knowledge).	Environmental restructuring Education	Information about health consequences. Information about social and environmental consequences. Instruction on how to perform the behaviour.	Develop educational resources (email and hard copies via mail).
Lack of time to implement. Perceived priority for the service.	Motivation- reflective (intentions, beliefs about capabilities).	Enablement Persuasion	Verbal persuasion about capability. Focus on past success. Discrepancy between current behaviour and goal. Social support.	Conduct educational outreach visits (online meetings, phone calls). Prepare and appoint GOGA Champion (email and online meeting).
Lack of awareness of alignment with COVID-19 Guidelines for NSW ECEC services and the National Quality Framework.	Motivation- reflective (Social /professional role and identify).	Enablement Education	Credible source. Information about social and environmental consequences.	Conduct educational outreach visits (online meeting). Develop education resources (email and hard copies via mail). Support from health promotion staff in local health district (centralized technical assistance).
Logistical arrangements: Including service staffing, service layout not conducive to outdoor play. Lack of specific plans to put the intervention into effect.	Capability –physical opportunity (Environmental context and resources).	Environmental restructuring Enablement	Restructuring the physical environment. Goal setting (behaviour). Problem solving. Action planning.	Conduct educational outreach visit (online meeting). Provide local technical assistance (online meetings and phone calls). Performance review and feedback (online meeting). Develop a formal implementation blueprint (online meetings).
Perceived lack of support from service managers/peers/ parents.	Opportunity-social (Social influences).	Persuasion	Social support (practical). Information about others’ approval.	Prepare and identify local GOGA Champions (email and online meeting). Develop education resources (email and hard copies via mail).
Unable to identify and attribute positive changes/outcomes to implementation efforts.	Motivation- reflection (Intentions, Beliefs about consequences).	Persuasion Enablement	Review behaviour goals. Self-monitoring of behaviour. Feedback on outcome of behaviour.	Audit and feedback (feedback summary (email), online meeting).

The research team then used the Theory and Techniques tool to select appropriate Behaviour Change Techniques (BCTs) to target the corresponding barriers [40]. Table 1 outlines the specific barriers mapped to the COM-B and TDF constructs, intervention function and the selection of BCTs to target the barriers. The Expert Recommendations for Implementing Change (ERIC) taxonomy was then used to select and describe implementation strategies to facilitate the delivery of the selected BCTs [41]. The final selection of implementation strategies included those that had evidence of effectiveness in ECEC or other parallel settings [26, 42], and selection were overseen by an expert group consisting of implementation and behavioural scientists, physical activity specialists, health promotion staff, representatives from early childhood organisations and health policy makers [41]. Finally, to determine suitability for scale up, the intervention strategies were also considered against the five domains of Part B of the Intervention Scalability Assessment Tool (ISAT) (fidelity and adaptation, reach and acceptability, delivery setting and workforce, implementation infrastructure and sustainability) [43], similar to that undertaken by Barnes and colleagues [44]. The research team also selected delivery modalities (email, online and/or via telephone) that were amenable for delivery at scale.

#### Implementation strategies

Following baseline data collection and randomisation, services allocated to the intervention arm will receive the implementation strategies described below, delivered over a 6 month period (T_1_-T_4_). The implementation strategies will be delivered by experienced Health Promotion Officers (HPO) employed by the LHD in the study region (centralized technical assistance). These staff hold tertiary qualifications related to nutrition, physical activity and health promotion. All staff have undertaken behaviour change training with an expert behavioural scientist to support delivery of the BCTs outlined in Table 1.

##### Identify and prepare champions [37]

The use of program champions can enhance implementation of EBPs through various mechanisms including trust-based relationships, role modelling and advocacy [45]. ECEC services will be contacted by the HPO via email and asked to nominate a GOGA Champion for the intervention. This may be the Service Manager or another existing staff member e.g. Room Leader, who will be responsible for supporting implementation of indoor-outdoor free play at the service. A position description for the Champion will be provided outlining the expectations for the role. This includes overseeing the development and implementation of an action plan (see c) and monitoring implementation progress. As part of the audit and feedback process, the nominated Champion will be supported by the HPO to reflect on changes to practice, identify positive implementation outcomes for Educators and children, and share these observations with staff and parents. Throughout the intervention, the Champion will also provide encouragement and recognition and/or further support to overcome indifference or behavioural challenges that may arise in regards to the intervention.

##### Develop and distribute educational materials [46, 47]

The development and distribution of educational materials is recommended to promote the evidence base for the EBP and provide guidance for effective practice [46]. HPO will provide the Service Manager and GOGA Champion with an electronic and hard copy information pack including a variety of educational materials regarding how the intervention aligns with ECEC accreditation standards and social distancing guidance by the NSW Department of Education for ECEC services. Other resources will include case studies from local services, fact sheets addressing common barriers, family newsletter snippets and “foot print” floor stickers to remind Educators and encourage children to “get active outside”. A GOGA newsletter for all ECEC staff will be distributed on four occasions throughout the intervention period and will include tips on overcoming common barriers to implementation (e.g. adverse weather, outdoor play resources, equipment and sustaining practice).

##### Conduct educational outreach visit(s)

Educational outreach visits used alone or in combination with other strategies may improve the implementation behaviour via the face to face provision of information required to change practice [48]. Initially (week 1, T_1_) a 30 min online meeting will be undertaken with the Service Manager and appointed GOGA Champion (and/or relevant room leader), facilitated by the HPO. The purpose of this meeting will be to orient the service to the implementation intervention (planned schedule of contacts with the service), provide and reinforce information on the EBP and sector guidelines, provide supporting information on other benefits of increasing outdoor free play, and discussions around how indoor-outdoor free play may complement existing service philosophy and priorities. An electronic action planning workbook (see c) will be provided and the steps to complete the workbook explained. In order to ensure the action plan has “all of service” input, the Service Manager and GOGA Champion will be asked to organise an information session for Educators at the service and identify a strategy to consult with other relevant staff. The information session may be delivered by the HPO (approx. 20 mins) or alternatively, the service may elect to receive a pre-recorded version of the information session presentation for staff to watch at their convenience. The Service Manager/Champion will guide what process to take to obtain wider input on the action plan from Educators. This could include a brainstorming session (approx. 20 mins), facilitated by the HPO at the conclusion of the delivered presentation, or the service may conduct this process independently as per their usual service procedures for consulting with staff. The service will be asked to complete these activities prior to week 8 of the intervention.

##### Develop a formal implementation blueprint

The development of a formal implementation blueprint (i.e. action plans) can be effective in broadening mindset about actions organisations could take to change current practices to successfully implement programs [49]. Additionally, an implementation blueprint has been shown to be an important effective modifier to audit and feedback strategies (i.e. performance feedback and review) [50].

The HPO will provide the Service Manager and GOGA Champion with an electronic action plan workbook at the first educational outreach meeting, and will discuss its intended use to guide service development of a plan to implement indoor-outdoor free play. The workbook includes: space to identify and document motivations and anticipated benefits of increasing outdoor free play opportunities, guidance around identifying suitable long and short term goals, an environmental checklist, problem solving tips and sample actions. The GOGA Champion will be asked to complete the workbook and final action plan prior to week 8 of the intervention.

##### Provide local centralized technical assistance

Organisations often require a support system to successfully implement new programs, and technical assistance provided by outside parties is key [51]. The HPO will provide local technical assistance at approximately three occasions throughout the 6 month intervention, via online meetings or phone. At approximately week 8 of the intervention, the HPO will arrange a meeting with the Service Manager and/or GOGA Champion to view and discuss the service action plan. The online meeting will take approximately 20–30 min, and will be an opportunity to ensure the action plan contains desired elements and appropriate timeframes for actions. The HPO will also provide positive reinforcement, facilitate reflection and provide problem solving advice and additional resources if required. The HPO will request an electronic copy of the final action plan to use as a reference during subsequent contacts.

The HPO will also undertake a 20 min phone support call with the GOGA Champion on two additional occasions (approximately week 10 and week 20) (T_2_ and T_3_). This will include reviewing progress with goals and actions, problem solving and setting any new goals or actions if required. This support will be tailored according to the preferences, needs and/or barriers of each service, and as such additional support calls or email support may occur at time points outside of those specified, and will be recorded as part of adaptations to the program.

##### Performance feedback and review

As recommended in a systematic review to optimise feedback provision [52], ECEC services will receive written and verbal feedback on their performance against baseline data at approximately week 12 (mid-way through the intervention, T_2_) via an online meeting or telephone call (duration approximately 30 min). The HPO will provide the GOGA Champion with a hard copy or electronic version of an observation tool to observe, record and reflect on both indoor and outdoor free play opportunities provided over a 5-day period. On completion, the GOGA Champion will submit the tool to their HPO. Based on this data, a written feedback report comparing the current provision of opportunities for outdoor free play with service goals and with the provision of outdoor free play at baseline will be provided. Verbal performance feedback and recognition of achievements will also be provided by phone or online meeting.

#### Control group

The delivery of all intervention components will be under the control of the research team, and will not be provided to comparison group services during the intervention period. ECEC services in the control group will receive ‘usual’ implementation support delivered as part of state-wide obesity prevention programs. This includes the provision of information and resources via a website and email contact, including factsheets and example policies and templates related to physical activity in general but not outdoor free play specifically. Additionally, COVID-19 safety protocols for the sector recommended that ECEC services provide additional time for outdoor only and indoor-outdoor free play. Nonetheless, data regarding ECEC’s exposure to such support and other potential sources of contamination will be assessed.

### Data collection and measures

#### Primary trial outcome: mean minutes ECEC services provide children with the opportunity for outdoor free play per week

The primary trial outcome is mean minutes of outdoor free play opportunities provided in care measured at baseline, 6-months (primary end point, T_5_) and 18-months following baseline (T_6_). This will be collected using a Free Play Record (FPR), adapted from existing ECEC measures of outdoor play [53, 54]. The Educator from the room targeted in intervention services will be asked to report on the time each free play session commenced and ended during their hours of operation, and report whether each session was held in the indoor only, outdoor only or indoor-outdoor environment, for 5 consecutive days. This will be defined as representing a typical week at the service. For example, it is to be completed on days where excursion or event which may disrupt your services usual routine are not planned. Where necessary, follow up phone calls or emails will be undertaken by the research team within a week of receiving the record to collect additional information needed to quantify total outdoor play. The FPR has been pilot tested with two ECEC services and reported to be feasible and acceptable (taking less than 5 mins/day for completion on average).

### Secondary outcomes

#### Proportion of time (minutes) ECEC services provide children with the opportunity for outdoor free play per week

The FPR will be used at baseline, 6-months and 18-months post baseline to calculate the percentage of total free play minutes services provide children with access to the outdoor environment during free play periods. Using the FPR, service Educators will report on the duration (minutes and/or hours) of indoor only, outdoor only, and/or indoor-outdoor play occurring during their hours of operation, for one week (5 consecutive days). This will be calculated as total outdoor only and indoor-outdoor routines over total available minutes for free play.

#### Proportion of services implementing the recommended indoor-outdoor free play routines per day for an entire week

The FPR will be used at baseline, 6- and 18-months post baseline to calculate the proportion of services implementing indoor-outdoor routines for the entire operational day (as per guideline recommendations). A service will be classified as implementing indoor-outdoor routines for the entire operational day if children are provided with the opportunity to access the outdoor environment during every session of free play (and therefore do not offer any free play exclusively indoors during the service hours of daily operation). If a service reports offering any free play exclusively indoors during the service hours of daily operation, they will be classified as not implementing indoor-outdoor free play routines.

#### Level of educator interaction with children during outdoor and indoor-outdoor free play

Increased implementation of outdoor free play routine has been shown to be associated with improve educator quality of intervention [55]. Hence, to assess potential impact of the intervention on educator interaction, trained research staff will observe interactions between Educators and children for one session of free play (morning or afternoon) on a single day scheduled with the service, at 6-months post baseline, using the movement environment rating scale (MOVERS). MOVERS assesses environments supporting children’s physical development and movement, with a focus on process quality, including children’s physical experience, and Educator practices [56]. MOVERS includes 11 items across 4 subscales; i) Curriculum, environment and resources for physical development (4 items); ii) Pedagogy for physical development (3 items); iii) Supporting physical activity and critical thinking (3 items); and iv) Parents/carers and staff (1 item) [56]. MOVERS has been designed to be worked through systematically (starting with item 1, then 2 and so on) and each item is scored on a 7-point scale (1 = inadequate, 3 = minimal, 5 = good, 7 = excellent). The total score and sub-scale scores are based on the average of the scores. Higher scores for the subscale ‘curriculum, environment, and resources for physical development’ indicate higher observed Educator engagement, prompts and facilitation of the space and resources [56], and has been shown to be positively associated with MVPA in pre-schoolers (β = 0.08 (95% CI: 0.01, 0.14)) [57]. MOVERS has been shown to have good test-retest reliability (weighted Kappa = 0.91; percentage agreement = 69–100%), internal consistency (Cronback’s α = 0.94), and concurrent validity (Spearman’s ρ = 0.57–0.87) [58].

### Other measures

#### ECEC characteristics

Data regarding the operational characteristics of ECECs, including service type, enrolment numbers, hours of operation, and the number of Educators employed at the service, will be collected via a baseline survey of ECEC Service Managers and Educators using items sourced from previous surveys of ECECs conducted by the research team [59]. The survey will also collect information regarding the characteristics of Service Managers including their highest level of relevant qualification, and length of employment within their current role as Service Manager. Ages of children in attendance at the service, and whether they come from a non-English speaking background will also be collected. Service location postcode will be used to assess socioeconomic status (SES) of the area and rurality.

#### Scalability outcomes

To measure potential scalability of the implementation intervention (GOGA), a number of outcomes outlined in the ISAT [60] will also be assessed at 6-months follow up using the following quantitative measures (described below) and additional qualitative interviews.

- *Acceptability:* At follow-up, intervention Service Managers and Educators will be invited to complete a post-intervention questionnaire and will be asked to report on the acceptability of the intervention using four-items based on the reliable and validated Acceptability of Intervention Measure (AIM) measures, developed by Weiner and colleagues [61].
- *Reach:* Reach will be measured as the number, proportion and representativeness of ECEC services who take part in the intervention, assessed via internal project records maintained by HPOs*.* The pre-recruitment telephone call will provide data on service characteristics for non-participating ECEC services.
- *Fidelity*: Fidelity of intervention delivery will be measured using monitoring instruments (online databases) within a secure web platform (REDCap), together with post-intervention questionnaires completed by intervention Service Managers, Champions and Educators, as well as internal project records maintained by HPOs.
- *Adaptation:* We will also track intervention delivery adaptations, including any adaptations to implementation strategies according to the FRAME-IS criteria [62] which is embedded within the intervention delivery monitoring instruments. HPOs will record adaptations as they occur under individual service records.
- *Sustainability*: An 18-month follow up is planned to assess the sustainability of the impact of the GOGA program [63]. Determinants of sustainability as outlined in the Integrated Sustainability framework will be assessed using measures developed by the research team at 6 and 12 months.
- *Implementation cost:* A cost consequence analysis will be conducted from the perspectives of the ECEC service and the health service. The costs and resource use for the intervention and usual care will be derived from intervention delivery monitoring instruments (staff and consumables), and Service Manager and Educator surveys. Additional costs in the intervention group are anticipated to be labour (implementation support), time and resource costs. Where data are unavailable, the basis for assumptions will be detailed. It is anticipated that a synthesis of other studies will not be required to derive appropriate assumptions. The reportable outcomes will be mean cost per service, mean incremental cost, and total cost to health providers used to calculate a composite score using the Incremental Cost Effectiveness Ratio (ICER). Sensitivity and scenario analyses will be undertaken to test the impact of changing key design features of the intervention and scale-up of the implementation model.

### Overall data management

Management of trial data will be in accordance with a data management protocol, which has been developed and approved by the project’s advisory group. Data will be stored in accordance with the requirements of all ethics committee. Data will only be accessible to investigators listed on the ethics application and statisticians.

### Analysis and sample size

Analyses will be performed using an intention to treat approach, with ECEC services as the unit of analysis. Separate analyses will be performed at each follow-up time point (6 and 18 months). Intervention effects on the primary trial outcome (at each follow-up time point) will be assessed using a linear mixed effects regression model, which will include fixed effects for the treatment group (intervention vs control), the baseline value of the outcome and variables that are prognostic of the outcome (service size and geographical region). Multiple imputations will be performed for ECEC services not providing follow up data in accordance with the recommendation by White and colleagues [64] and presented as the primary analysis. Continuous secondary outcomes will be analysed using a linear mixed effects regression model similar to the primary outcome. Dichotomous secondary outcomes will be assessed using a logistic regression model. A sensitivity analysis will be undertaken, adjusting for service characteristics that appear imbalanced between groups at baseline (if this is present). Additionally we will plan to undertake a subgroup analysis examining potential intervention effect among services with the lowest baseline outdoor free play opportunities (specified as the lowest third quartile).

For the primary outcome (mean minutes of opportunities for outdoor free play), a sample of 100 ECEC services will enable us to detect an absolute difference of 25.34 min/day in the time children have the opportunity to spend in outdoor environments during free play assuming a standard deviation of 43 min with 80% power and an alpha of 0.05 based on previous research [65]. An additional 27 min of outdoor free play time will increase total outdoor free play time to > 1 h for many services, resulting in an additional 10 min of MVPA [13]. An increase of 10 min in MVPA in children aged 3 to 6 years has been found to have clinically significant beneficial effects on fat mass and peak bone mass [66, 67].

For the secondary outcome (the proportion of services implementing the recommended indoor-outdoor free play routines per day for an entire week), a sample of 100 ECEC services (50 per group) will enable us to detect an absolute difference of 27.50%, assuming that 31% of control services implement an indoor-outdoor program as recommended, with 80% power and an alpha of 0.05 based on previous research [65].

#### Research trial governance

This study has employed a research co-production approach in the design of its aims, evaluation approaches and intervention design. An advisory group consisting of expert implementation and physical activity researchers, policy makers, health practitioners, and representatives from early childhood organisations will oversee all aspects of the trial. A project team consisting of research staff and health promotion practitioners will develop and operationalise implementation strategies and data collection components of the trial according to study protocol. The lead author together with the advisory group will oversee the project dissemination plan including all publications and reports to stakeholders. Authorship will conform to the International Committee of Medical Journal Editors (ICMJE) guidelines.

#### Trial discontinuation or modification

It is not anticipated that any events would occur that would warrant discontinuing the trial. Changes in government mandate related to COVID-19 restrictions may require modification to data collection or intervention delivery modality. Any unforeseen adverse events will be reported to HNE HREC (primary approval committee) and appropriate action taken to address the event. The trial registration record will be updated with any protocol modifications and any deviations from original protocol will be reported when publishing trial outcomes.

## Discussion

This trial is one of few RCTs to assess the effectiveness of strategies to increase the implementation of evidence-based physical activity practices at scale in ECEC services in Australia. This intervention applies an evidence-based theory mapping approach to intentionally select strategies and intervention modalities that are amenable to scaling up. If effective, this trial will provide a strategy that could be used to deliver support to increase evidence-based physical activity programs to increase the impact of health promotion programs in this setting.

## Data Availability

Study is a study protocol and no data is generated as part of the manuscript. Data sharing is not applicable to this article as no datasets were generated or analysed during the current study.

## Abbreviations

AIM: Acceptability Intervention Measure
BCTs: Behaviour Change Techniques
BCW: Behaviour Change Wheel
COM-B: Capacity, Opportunity & Motivation Models of Behaviour
CPH: Centre for Population Health
EBP: Evidenced Based Practice
ECEC: Early Childhood Education and Care
ERIC: Expert Recommendations for Implementing Change
FPR: Free Play record
GOGA: Get Outside Get Active
HNE: Hunter New England
HPO: Health Promotion Officer
HREC: Human Research Ethics Committee
ICER: Incremental Cost Effectiveness Ratio
ICMJE: International Committee of Medical Journal Editors
ISAT: Intervention Scalability Assessment Tool
LHD: Local health District
MVPA: Moderate-to-Vigorous Physical Activity
NSW: New South Wales
RCT: Randomised Control Trial
SES: Socioeconomic Status
TDF: Theoretical Domains Framework
UK: United Kingdom
US: United States
